# Occurrence of Heat-Resistant Mold Ascospores in Pineapple and Sugarcane Field Soils in Thailand

**DOI:** 10.1155/2023/8347560

**Published:** 2023-07-27

**Authors:** Thanapoom Maneeboon, Somsiri Sangchote, Ratchanee Hongprayoon, Chananya Chuaysrinule, Warapa Mahakarnchanakul

**Affiliations:** ^1^Center for Agricultural Biotechnology, Kasetsart University, Kamphaeng Saen Campus, Nakhon Pathom 73140, Thailand; ^2^Center of Excellence on Agricultural Biotechnology (AG-BIO/MHESI), Bangkok 10900, Thailand; ^3^Scientific Equipment and Research Division, Kasetsart University Research and Development Institute (KURDI), Kasetsart University, Bangkok 10900, Thailand; ^4^Department of Plant Pathology, Faculty of Agriculture, Kasetsart University, Bangkok 10900, Thailand; ^5^Department of Plant Pathology, Faculty of Agriculture at Kamphaeng Saen, Kasetsart University, Kamphaeng Saen Campus, Nakhon Pathom 73140, Thailand; ^6^Department of Food Science and Technology, Faculty of Agro-Industry, Kasetsart University, Bangkok 10900, Thailand

## Abstract

Heat-resistant molds (HRMs) are important spoilage fungi of heat-processed fruit products worldwide. Ascospores of HRMs are widely distributed in the soil in which fruits are grown and are often found associated with raw fruit materials. To date, there is little available information on the distribution of HRMs in the soil and on their heat resistance. Thus, this study determined the presence and characterized the heat resistance of HRMs in soil samples from pineapple and sugarcane fields in Thailand. HRMs were detected in all soil samples, and the most dominant species was *Aspergillus* with 50–99.2% relative abundance. Other isolates, in descending order of frequency, were *Penicillium*, *Talaromyces*, *Hamigera,* and *Paecilomyces*. Then, 100 representative HRM isolates were identified based on a combination of morphological characteristics and ITS sequences. They were classified into 5 genera and 24 species. The heat resistance of ascospores aged 30 days produced by selected HRMs was qualitatively determined in a glucose-buffered solution. Based on their log reductions after heat shock at 75°C for 30 min, they were classified as less, moderately, or highly heat-resistant ascospores. HRMs belonging to *A. chevalieri*, *A. denticulatus*, *A. siamensis*, *A. laciniosus*, *A. fennelliae*, *A. spinosus*, *Paec. niveus*, *H. pallida,* and *T. macrosporus* produced high heat-resistant ascospores. In addition, soil physicochemical properties significantly influenced the prevalence of HRMs, depending on the fungal genus. The thermal resistance of ascospores was significantly and positively correlated to available phosphorus, whereas it was negatively correlated to soil pH. The results of this study confirmed the presence of HRMs in soils and potential HRM contamination, especially in fruits growing in acidic or high-nutrient soils, or both.

## 1. Introduction

Heat-resistant molds (HRMs) have been associated with the spoilage of pasteurized fruit-based products because HRMs can survive certain heat treatments used in food processing. The fungi that are broadly distributed in the food and beverage processing environment are typically killed by wet heating and inactivated by a pasteurization temperature of 70°C for 10 min [1]. The heat resistance of HRMs has been attributed to the formation of thick-walled sexual spores called ascospores [2], defined as being capable of surviving temperatures at or above 75°C for at least 30 min [3]. Especially in acidic fruit-based products, the thermal treatments that are typically sufficient to inactivate most enzymes, microbial, and fungal vegetative cells do not affect the ascospores of HRMs [4]. Ascospores of heat-resistant fungi can survive temperatures in the range of 55–95°C [5].

Ascospores of HRMs are dormant and germinate after a strong external shock, such as heating or exposure to some chemicals [6]. The principal HRMs belong to the *Aspergillus* (with *Neosartorya*-morph), *Talaromyces*, *Paecilomyces* (with *Byssochlamys*-morph), *Penicillium* (previously classified as *Eupenicillium*), and *Hamigera* [7]. Under certain conditions, some species of HRMs may produce mycotoxins that are toxic to consumers. For example, certain strains of *A. fischeri* (*N. fischeri*) have been reported to produce verruculogen, terrein, and fumitremorgins. Some species of *Paecilomyces* may produce patulin, byssotoxin A, and byssochlamic acid [8].

Ascospores of HRMs are widely spread in soils, which are the main source of these fungi. In addition, ascospores are often found associated with fresh fruits, particularly those that come into contact with soil or may be contaminated by rain splash [9]. Furthermore, HRMs may be carried to food processing plants by dust and on fruit surfaces, resulting in contamination of the processing areas and finished foods [10]. HRMs have been reported to cause spoilage in various fruits and fruits products, including fruit yogurt, ice cream with fruits, fruit juices [11], concentrated apple juice [12], strawberry semi-finished product, and sweetened beverage [13]. However, there appear to be no published studies on the incidence of HRMs in pineapple and sugarcane field soils. Therefore, the objectives of this study were to determine the prevalence of HRMs in the soils where pineapple and sugarcane are grown and to characterize the heat resistance of the ascospores of some HRM isolates.

## 2. Materials and Methods

### 2.1. Soil Samples

In total, 12 soil samples were randomly collected, with 9 from pineapple fields in Chonburi and Kamphaeng Phet provinces and 3 from sugarcane fields in Sa Kaeo and Prachin Buri provinces, Thailand. Approximately 0.5 kg soil samples were taken from the 0–5 cm depth and kept in sterile plastic bags. Three to five samples were collected at each site and then mixed into one soil sample to represent one location. Soil samples were placed in sterilized polyethylene bags and transported to the laboratory in a cooler box. Soils were passed through a 2 mm sieve to remove stones and plant materials, stored at −20°C, and processed within 48 h.

### 2.2. Isolation of HRMs

The Petri dish method proposed by Rico-Munoz et al. [14] was used for isolation of HRMs, with some modifications. Briefly, 10 g of each soil sample was weighed out aseptically and transferred into 240 ml sterile distilled water in a zippered plastic bag. Then, the bags were sealed and heated in a circulating water bath at 75°C for 30 min. After heating, each sample suspension was allowed to cool to 55°C; then, 250 ml of soil suspension was aseptically added to an equal volume of warm (approximately 55°C) double-strength malt extract agar (MEA) containing 50 mg/l rose bengal and 100 mg/l chloramphenicol. The mixture was thoroughly mixed and dispersed into 150 mm Petri dishes. The plates were loosely sealed in a plastic bag to prevent drying and incubated at 30°C for at least 14 days. After the incubation period, representative types of fungal colonies were counted; the results were reported as colony-forming units per gram soil (CFU/g). The representative fungal colonies were picked up and then subcultured on potato dextrose agar (PDA).

### 2.3. Morphological Identification

Initially, fungi were categorized based on morphospecies using their colony characteristics when grown on MEA, oatmeal agar (OA), and dichloran glycerol (DG18) agar incubated at temperatures of 25 and 35°C for 7 days [15]. Fungal morphology and physiology were studied for identification at both the genus and species levels using macroscopic and microscopic characteristics, including colony morphology, hyphal structure, and spore arrangement, following the described methods for *Aspergillus* [16], *Talaromyces* [17], *Hamigera* [18], *Penicillium* [19], and *Paecilomyces* [20].

### 2.4. Molecular Identification

#### 2.4.1. Isolation of Fungal DNA

Genomic DNA was extracted from fungal mycelia as described by Umesha et al. [21] with some modifications. Representative isolates of each morphospecies were grown on PDA for 7 days. Fungal mycelia (50 mg) were added into 1.5 mL tube with glass beads and frozen at −20°C for 60 min. Then, 600 *μ*L of lysis buffer (1% sodium dodecyl sulfate, 50 mM Tris-Cl (pH 8.0), 50 mM ethylenediaminetetraacetic acid, and 2% mercaptoethanol) was added, the mycelia were crushed with a plastic pestle and incubated at 65°C for 30 min. After centrifugation at 12,000*xg* at 4°C for 5 min, genomic DNA was extracted from the supernatant by adding equal volume of phenol/chloroform (1 : 1, v/v) and the samples were centrifuged at 12,000x*g* at 4°C for 10 min. The supernatant was collected and extracted again using equal volume of chloroform/isoamyl alcohol (24 : 1, v/v). Then, samples were centrifuged at 12,000x*g* at 4°C for 10 min, and the supernatant was transferred into a new tube. The DNA was precipitated by adding 0.7 and 0.1 volumes of chilled isopropanol and sodium acetate (0.3 M), respectively. After centrifugation at 12,000x*g* at 4°C for 2 min, the DNA pellet was washed twice with 200 *μ*L chilled absolute ethanol, after which the tubes were centrifuged at 5,000x*g* for 2 min. The DNA was air dried at room temperature, resuspended in 50 *μ*L of deionized water, and stored at −20°C for future use.

#### 2.4.2. Polymerase Chain Reaction (PCR)

The PCR amplification of genomic DNA was performed using universal primers for fungal DNA at the internal transcribed spacers (ITSs): ITS1 (5′TCCGTAGGTGAACCTGCGG3′) and ITS4 (5′TCCTCCGCTTATTGATAT GC3′) [22]. The PCR reaction was carried out as described by Anukul et al. [23]. A 25 *μ*l sample of the reaction mixture contained 1 × ReadyMix with Mg^2+^ (KAPA2G Fast HotStart DNA Polymerase, Kapa Biosystems, South Africa), 1 *μ*l of 10 pmol of each primer, and 20 ng of DNA. The PCR conditions used to amplify the target genes were an initial denaturation step of 3 min at 95°C, 35 cycles (each 15 s) for denaturation at 95°C, 15 s for annealing at 60°C, and 10 s for the extension step at 72°C. The final extension was carried out for 1 cycle at 72°C for 1 min.

#### 2.4.3. Sequence Analysis

PCR products (500–600 kb) were sequenced using the barcode-tagged sequencing technique by U2Bio (Thailand) Co., Ltd. The sequence of each fungal isolate was compared with partial ITS sequences available in the National Center for Biotechnology Information (NCBI) database using the Basic Local Alignment Search Tool for Nucleotide Sequences (BLASTN; https://blast.ncbi.nlm.nih.gov/Blast.cgi) to identify these fungal species. Sequences were aligned using Muscle in the MEGA version 11 software [24]. Phylogenetic trees of the obtained and reliable reference sequences were generated using the neighbor-joining method with 1,000 iterations of bootstrapping. Sequences derived in this study were deposited in the GenBank nucleotide database under the accession numbers OP480880-OP480981.

### 2.5. Ascospores Preparation

The fungal isolates were grown on PDA for 7 days at room temperature. Fungal spores were harvested by adding 10 ml of 0.1% Tween 80 to the surface of a fungal colony. Then, 0.1 ml of spore suspension was spread onto MEA and incubated at 30°C for 30 days [25]. Ascospores were collected by flooding with sterilized 0.1% Tween 80. After centrifugation at 4,000x*g* for 10 min, the supernatant was discarded and 5 ml of sterile 0.1% peptone water was added to the pellet and mixed. Washing with 0.1% peptone water was performed three times. To separate the ascospore cluster, sterile glass beads were added into the spore suspension and mixed for 2 min using a vortex mixer. Then, the ascospore suspension was sonicated for 10 min and filtered through a sterile cotton layer. The filtered ascospore suspensions were heated at 65°C for 15 min in a water bath to kill any fungal vegetative structures before storing the suspension in a 50 ml sterilized falcon tube containing sterile glass beads at −20°C until use.

### 2.6. Determination of Viable Ascospores

To determine the initial ascospore concentration, the ascospore suspension was heated at 75°C for 5 min to activate the dormant ascospores. Then, the ascospore suspension was adequately diluted and spread onto PDA containing 50 mg/L rose bengal and 100 mg/L chloramphenicol and incubated at 30°C. Enumeration was performed after incubation for 7 days.

### 2.7. Screening of Heat-Resistant Isolates

Ascospores produced by the HRMs isolates were investigated for their thermal resistance characteristic using the heat shock method in a glucose-buffered solution (12.5°Brix, pH 3.6 adjusted with 0.67 M tartaric acid) [22]. Each ascospore suspension was diluted 1 : 10 with a glucose-buffered solution to obtain a final ascospore concentration of 10^4^–10^5^ ascospores/ml. A 3 ml sample of the diluted ascospore suspension was poured into a polythene plastic bag. Then, the plastic bag was sealed to exclude any air inside and plunged into a water bath equipped with a thermometer. The mixture was treated at 75°C for 30 min. After the heat treatment, the plastic bag was rapidly cooled on ice. An appropriate decimal dilution was spread on PDA, as described above. Colonies were counted after incubation at 30°C for up to 7 days. The suspension of ascospores was heat activated at 75°C for 5 min to count the initial number of ascospores. Heat resistance was expressed as a log reduction as follows:

(1) $$\begin{array}{c} \mathrm{Log}\;reduction={\mathrm{Log}}_{10}\left(\frac{N_{5}}{N}\right)\text{,} \end{array}$$

where *N*_5_ is the initial number (CFU/ml) of ascospores as the control and *N* is the number of surviving ascospores (CFU/ml) after heat shock at 75°C for 30 min.

HRM strains were classified according to their survival after heating, using the following criteria. Strains with a reduction in surviving ascospores of more than 1 log were classified as less heat resistant. Those exhibiting a reduction in surviving ascospores of 0-1 log were classified as moderately heat resistant, while strains with a reduction of less than 0 log were classified as highly heat-resistant [26, 27].

### 2.8. Determination of Soil Physicochemical Properties

The moisture content of the soil was determined using the standard method of Black [28]. After mixing the soil with water (1 : 5 ratio), the pH and conductivity of the suspension were measured using a pH meter (EUTEC pH700, Eutech Instruments Pty Ltd., Singapore) and a conductivity meter (EUTECH CON700, Eutech Instruments Pty Ltd., Singapore), respectively. Total organic matter was determined based on oxidation with potassium dichromate and titration with ferrous ammonium sulfate [29]. The amount of available phosphorus in the soil was determined according to the Bray II method [30] and measured using a spectrophotometer (UV-1800, Shimadzu, Japan). The physicochemical properties of the soil analyzed in this study are presented in Table 1.

### 2.9. Statistical Analysis

All experiments were carried out in duplicate. The mean and standard error of all treatments were determined using the Microsoft Excel software. Principal component analysis was analyzed using the GraphPad Prism 9.0.0 Trial version software (GraphPad Software, Inc., USA).

## 3. Results and Discussion

### 3.1. Incidence of HRMs in Soil

**Figure 1:**
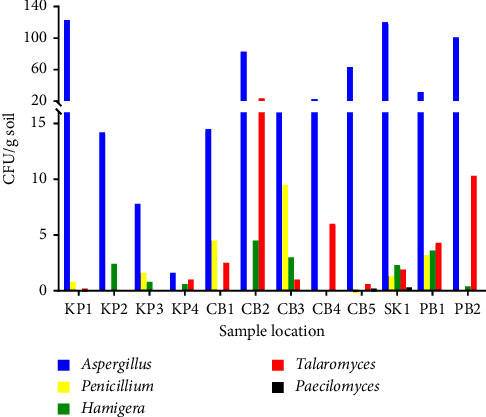
Total fungi count from soils with different soil sample locations. Each value represents the mean of two replicates.

The occurrence and distribution of HRMs in the soil samples from sugarcane and pineapple fields, as well as the predominant fungal species in each site, are shown in Figure 1. HRMs were detected in all soil samples. The distribution of HRMs in the soil varied from 3.2 to 125.7 CFU/g soil according to the sampling site. The largest population was in the soil from the sugarcane field in Sa Kaeo (SK1), whereas the smallest population was in the pineapple field in Kamphaeng Phet (KP1).

**Figure 2:**
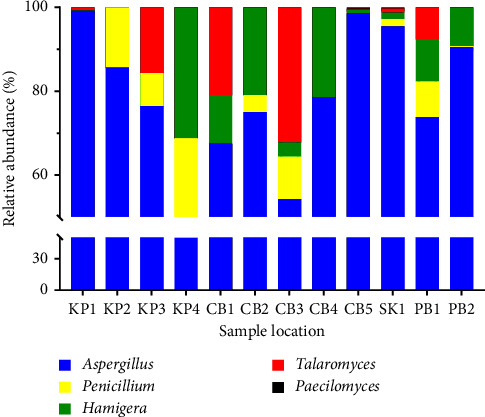
Relative abundance of different fungi at the genus level in different soil sample locations.

Based on their morphological characteristics (color and texture of the colony and color and formation of conidia), members of the *Ascomycota* were the major active fungi in the heat-treated soils and could be categorized into 5 different morphospecies. As shown in Figure 2, variation in the fungal diversity at the genus level was observed. Fungal genera with a relative abundance of more than 5% were considered as dominant species [31], which applied to 4 genera: *Aspergillus*, *Penicillium*, *Talaromyces,* and *Hamigera*. The genus *Aspergillus* was the most abundant and was identified in all soil samples, with relative abundance values in the range 50–99.2%. The other three main genera were *Penicillium* (0.2–32.2%), *Talaromyces* (0.2–31.3%), and *Hamigera* (0.4–18.7%) that were in some soil samples. In addition, *Paecilomyces* was detected, but it had the lowest relative abundance (0.2–0.3%). It was identified only in soil samples from Chonburi (CB5) and Sa Kaeo (SK1). Our results agreed with other findings in the similar studies of HRMs in forest and garden soils, for which 90–100% of the analyzed soil samples contained HRMs with high fungal loadings, with the most common being *Aspergillus* (with *Neosartorya*-morph), in the Slovak Republic [32] and Nigeria [33].

### 3.2. Species Identification

In the present study, 100 representative fungal isolates were selected from each different morphotype and identified at the species level based on the combination of morphological characteristics and ITS rDNA sequence analysis. The results from the BLAST analysis confirmed that all observed fungal isolates belonged to the *Ascomycetes*. Overall, 5 fungal genera and 20 fungal species were identified.

**Figure 3:**
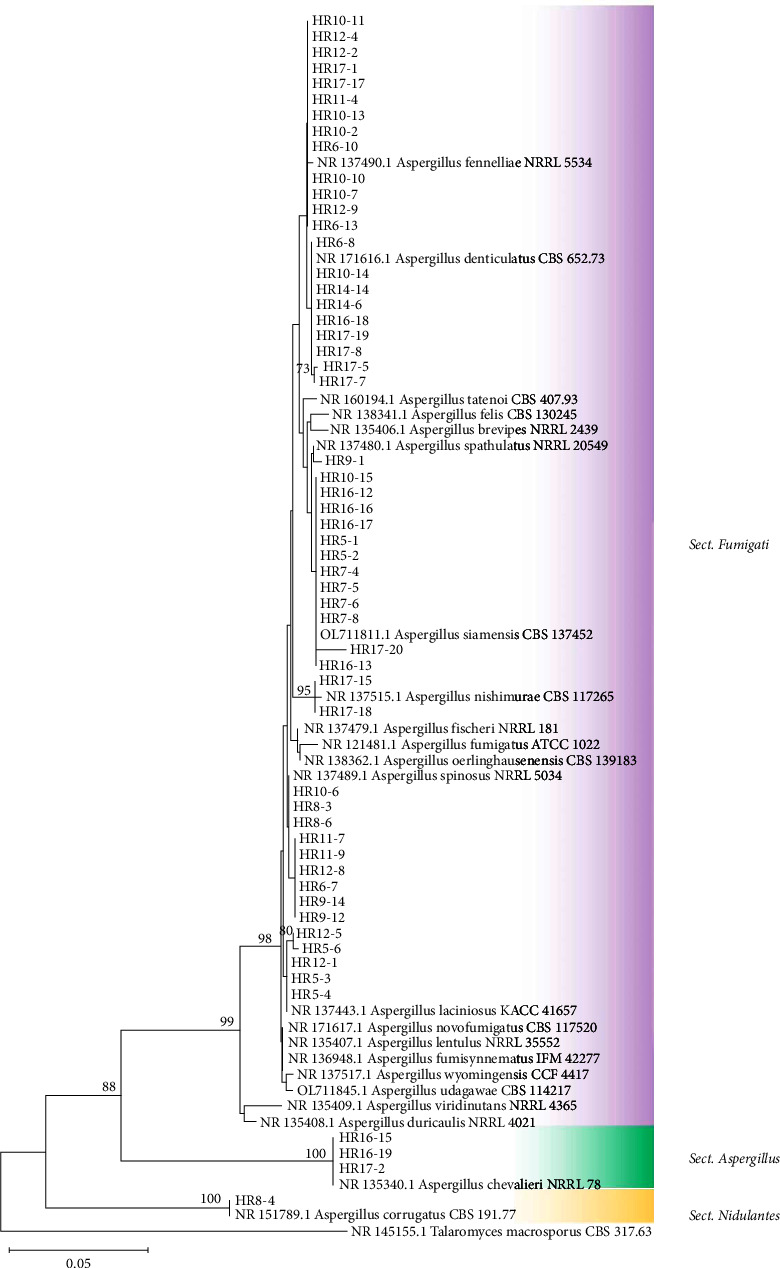
Phylogenetic tree reconstructed using neighbor-joining method based on ITS sequences of *Aspergillus* species with Kimura-2 parameter model. *Talaromyces macrosporus* is the outgroup.

Most heat-resistant species of *Aspergillus* belong to the section *Fumigati*, which are known as one of the most frequently identified and abundant species in a variety of soils [34]. In the present study, there were 55 isolates belonging to 9 species representing 3 sections. The phylogenetic analysis of the *Aspergillus* isolates obtained in this study and reference sequences resulted in the tree are shown in Figure 3. At the section level, the *Aspergillus* section *Fumigati* (92.72%) was dominant, followed by the section *Aspergillus* (5.55%) and the section *Nidulantes* (1.85%). Nine species of *Aspergillus* were identified, namely, *A. fennelliae* (*n* = 13), *A. siamensis* (*n* = 12), *A denticulatus* (*n* = 9), *A. spinosus* (*n* = 9), *A. laciniosus* (*n* = 5), *A. chevalieri* (*n* = 3), *A. nishimurae* (*n* = 2), *A. spathulatus* (*n* = 1), and *A. corrugatus* (*n* = 1). Even though *A. fischeri* is one of the most frequently isolated species from soil and heat-processed foods [35, 36], it was not isolated from any soil sample. This result was not consistent with the study by Eamvijarn et al. [37] in which the most abundant *Aspergillus* section *Fumigati* found in agricultural soils in Thailand was *A. spinosus* (31.8%), followed by *A. fischeri* (16.5%).

**Figure 4:**
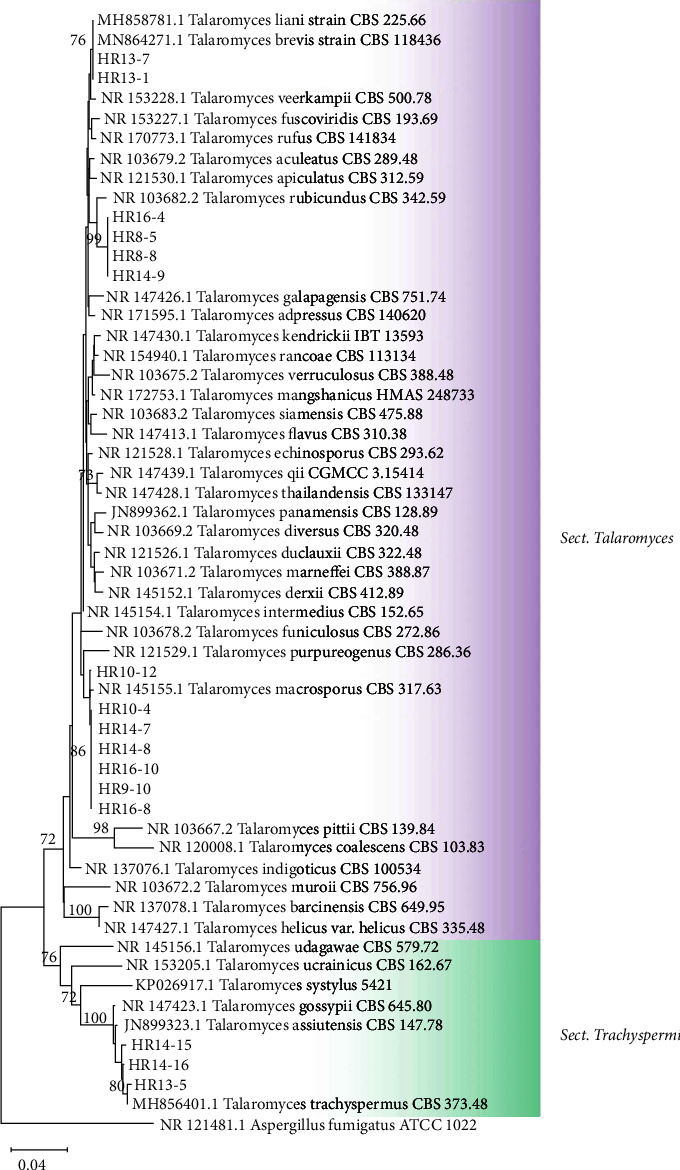
Phylogenetic tree reconstructed using neighbor-joining method based on ITS sequences of *Talaromyces* species with Kimura-2 parameter model. *Aspergillus fumigatus* is the outgroup.

**Figure 5:**
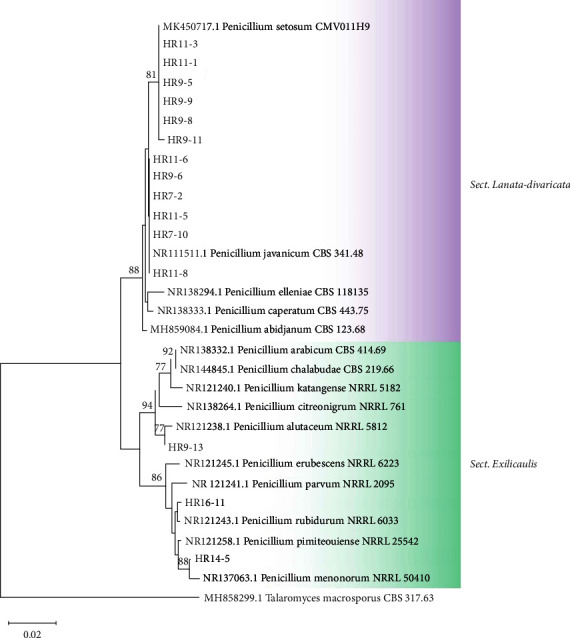
Phylogenetic tree reconstructed using neighbor-joining method based on ITS sequences of *Penicillium* species with Kimura-2 parameter model. *Talaromyces macrosporus* is the outgroup.

Sixteen isolates of *Talaromyces* were classified into 2 sections: *Talaromyces* and *Trachyspermi* (Figure 4). Thirteen isolates were identified as belonging to the section *Talaromyces*: *T. macrosporus* (*n* = 7), *T. rubicundus* (*n* = 4), and *T. brevis*/*T. liani* (*n* = 2). Three strains of the section *Trachyspermi* showed similarity to *T. trachyspermus*. In addition, our study indicated that 15 isolates of *Penicillium* species were grouped into 2 sections, namely, section *Lanata‐divaricata* and *Exilicaulis* (Figure 5). The phylogenetic analysis demonstrated that within the section *Lanata‐divaricata*, 6 isolates were identified as *P. javanicum*, while 6 others were classified as *P. setosum*. For the section *Exilicaulis, P. alutaceum*, *P. menonorum,* and *P. rubidurum* were isolated only once. Supporting our results, HRMs belonging to *Talaromyces* and *Penicillium* have been detected in fruit and fruit products with low frequency [22]. The major *Talaromyces* species, such as *T. macrosporus*, *T. flavus*, *T. trachyspermus,* and *T. bacillisporus*, are generally associated with spoiled fruit products [36, 38]. *Penicillium* species are widely distributed in soil, indoor environments, and food products [39]. For example, *P. javanicum* (*E. javanicum*), which is a fast-growing filamentous fungus, has also been isolated from a processed fruit [40, 41].

**Figure 6:**
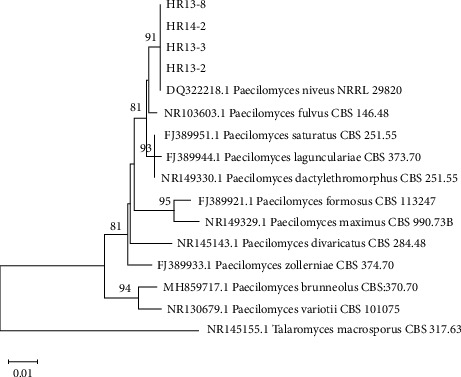
Phylogenetic tree reconstructed using neighbor-joining method based on ITS sequences of *Paecilomyces* with Kimura-2 parameter model. *Talaromyces macrosporus* is the outgroup.

**Figure 7:**
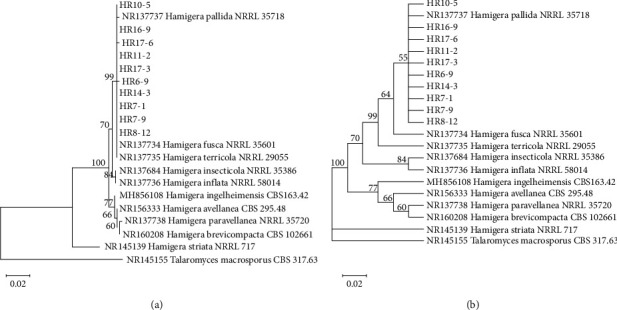
Phylogenetic trees reconstructed using neighbor-joining method based on ITS sequences of *Hamigera* species with Kimura-2 parameter model; (a) consensus tree and (b) 55% condensed tree. *Talaromyces macrosporus* is the outgroup.

In the present study, we found that two fungal genera belonging to the *Paecilomyces* and *Hamigera* were uncommon and represented by one species each. Based on the ITS dataset of 11 reference sequences of *Paecilomyces*, 4 isolates of *Paecilomyces* were clearly identified as *Paec. niveus* (Figure 6). In the case of *Hamigera*, phylogenetic analysis based on ITS sequences of 10 isolates obtained from the present study and the reference *Hamigera* species revealed that they grouped with *H. terricola*, *H. fusca*, and *H. pallida* (Figure 7(a)). However, the identity of certain *Hamigera* isolates remained ambiguous based on the ITS locus sequencing. Thus, the condensed phylogenetic tree was used to clarify the identity. As shown in Figure 7(b), it was observed that they formed a cluster with *H. pallida*. In general, the *Paecilomyces* species is abundant in soil and is recognized as an important spoilage mold of thermally treated fruit products [42]. In contrast to our findings, Luangsa-ard et al. [43] reported that the predominant species of *Paecilomyces* in forest soils in Thailand was *Paec. variotii*. Generally, *Hamigera* species are uncommon heat-resistant species due to their infrequent occurrence in raw fruit materials. Consistent with our findings, there have been few reported studies, with only the occurrence of *H. striata* isolated from frozen blueberries [44] and of *H. avellanea* isolated from strawberry semi-finished products [13].

### 3.3. Characterization of Thermal Resistance of HRMs

**Figure 8:**
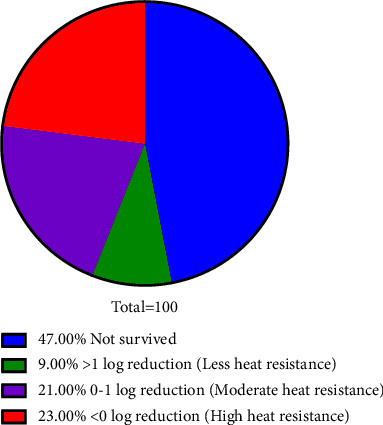
Heat resistance of ascospores produced by HRMs isolated from field soils.

Ascospores produced from the 100 HRM isolates suspended in a glucose-buffered solution (12.5°Brix, pH 3.6) were treated with heat shock at 75°C for 30 min. This suspending solution simulated the composition of fruit juice. The thermal resistance of the HRM isolates was categorized by determining a logarithmic viability reduction. The results of the survival of ascospores for each isolate after heat treatment calculated as the log reduction (log *N*_5_/*N*) are shown in Figure 8. In this study, a variation in the thermal resistance of HRM ascospores was observed and classified into 4 groups. We found that 47 isolates were not able to survive the heat shock treatment, while 53 isolates did survive with differing levels of thermal resistance. Among the heat-resistant isolates, 9 showed a reduction of greater than 1 log, indicating the ascospores of this group were less resistant to thermal treatment. The ascospores of the 21 isolates that were inactivated from a reduction of 0-1 log after heat treatment at 75°C were defined as moderately heat resistant. Some strains exhibited activation after heat shock, indicating they were the most heat resistant. An increase in the number of surviving ascospores after heat treatment (<0 log reduction) was exhibited by 23 isolates.

**Figure 9:**
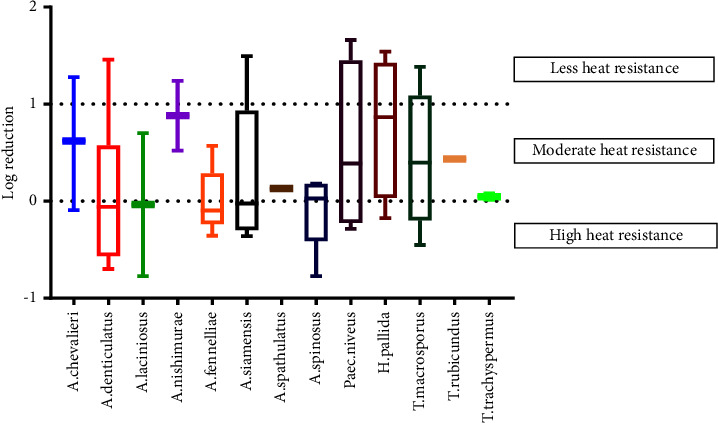
Box plots representing variability of log reduction (log *N*_5_/*N*) of ascospores produced by 13 HRM species that survived heat treatment at 75°C for 30 min in a glucose-buffered solution (12.5°Brix, pH 3.6). Horizontal lines within the boxes represent medians, box bounds at 25th and 75th percentiles, while the whiskers denote 10th and 90th percentiles.

Figure 9 presents the range and the median values for the log reductions of various HRM species after heat treatment. In summary, 4 of the 5 fungal genera survived the heat-shock treatment (*Aspergillus*, *Talaromyces*, *Hamigera*, and *Paecilomyces*), while *Penicillium* failed to produce heat-resistant ascospores. Considering the heat-resistant species from these 4 genera, 13 of the 20 HRM species exhibited thermal-resistant characteristics. The HRM isolates were grouped based on the heat resistance of their ascospores into less heat resistant, moderately heat resistant, and highly heat resistant. In general, we found variability in the heat resistance of ascospores between and within HRM species. Ascospores produced by various strains of *A. chevalieri*, *A. denticulatus*, *A. siamensis*, *Paec. niveus*, *H. pallida*, and *T. macrosporus* showed a large variation in heat resistance from less to highly resistant. Ascospores produced by *A. laciniosus*, *A. fennelliae*, and *A. spinosus* strains were highly and moderately heat resistant, while strains of *A. spathulatus*, *T. trachyspermus*, and *T. rubicundus* were classified as moderately heat resistant. In addition, ascospores of *A. nishimurae* were less and moderately heat resistant.

Differences in the thermal-resistant characteristics have been used for the screening of the most heat-resistant microorganisms [45, 46]. Variability has been reported in the thermal resistance of ascospores among different fungal strains even though grown and tested under similar conditions for *Paecilomyces* species and *A. fischeri* [25, 36]. Variation in the heat resistance of ascospores formed by different strains belonging to the same species is possibly due to intraspecific biodiversity [47, 48]. In addition, heat-resistant ascospores were not observed in the culture of some HRM species. This might have been because they have a heterothallic mode that needs the mating of two strains to produce heat-resistant ascospores, such as *Paec. variotii*, *A. fumigatus*, *A. fennelliae*, *A. spathulatus*, and *A. nishimurae* [49].

In the present study, highly heat-resistant ascospores were produced by strains of *Paec. niveus*, *H. pallida*, *T. macrosporus*, *A. laciniosus*, *A. spinosus*, and *A. chevalieri*. At the tested temperature, the number of emerging colonies of the most heat-resistant isolates increased when the heating time was extended. The number of colonies is expected to increase until reaching the population of ascospores and to decrease after prolonged heating [50]. The high heat resistance of these species has been published elsewhere [13, 51, 52]. There has been no reported evidence of the spoilage of fruit products caused by other highly heat-resistant species: *A. denticulatus*, *A. siamensis,* and *A. fennelliae*. All strains of *A. spathulatus*, *T. rubicundus,* and *T. trachyspermus* produced ascospores exhibiting moderate heat resistance, while *A. nishimurae* produced less to moderately heat-resistant ascospores. As far as we know, only *T. trachyspermus* has been investigated for heat inactivation [22].

### 3.4. Relationship of HRMs Species with Physicochemical Properties of Soil

**Figure 10:**
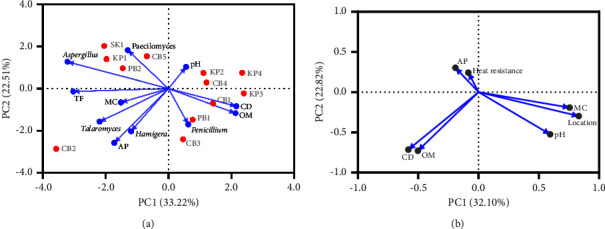
Principal component analysis (PCA) based on correlation matrix of soil physicochemical properties and characteristics of HRMs isolated from soils: (a) PCA score plot of the first two principal components (PC1 and PC2) for HRMs at genus level isolated from soil samples and (b) PCA loading plot of soil properties and log reduction (log *N*_5_/*N*) of ascospores for PC1 and PC2.

The relationships between the occurrence of HRMs species with soil physical and chemical properties were determined based on principal component analysis (PCA), as shown in Figure 10(a). The first two principal components explained 55.73% of the variation in the distribution of HRM species. Total fungi (TF) had strong negative loadings on PC1. *Aspergillus* and *Talaromyces* had a negative correlation with PC1. *Paecilomyces* was positively correlated to PC2, while *Penicillium* and *Hamigera* were negatively correlated to PC2. In addition, PCA revealed the relationship between the abundance of HRMs with soil properties. It was found that *Talaromyces* and *Hamigera* were significantly and positively correlated with moisture content (MC) and available phosphorus (AP), while they showed a significantly negative correlation with soil pH. *Aspergillus*, *Paecilomyces*, and total fungi (TF) were significantly and negatively correlated with conductivity (CD) and organic matter (OM).

As the population of HRMs was affected by the variation in soil physical and chemical properties, we investigated which soil properties were significantly correlated with the heat resistance of HRMs (inverse of the log reduction value). As mentioned, smaller log reduction values indicate greater resistance to heat by the ascospores. In the PCA analysis (Figure 10(b)), the first two principal components explained 54.92% of the total variation in the heat resistance of ascospores produced from various strains of HRM. We observed that MC had a positive correlation with PC1, while the heat resistance of ascospores had strong positive loadings on PC2. Furthermore, the PCA results clarified the relationship between the thermal resistance of ascospores and soil properties. We observed that the heat resistance of ascospores was strongly and positively correlated with AP, while it was significantly negatively correlated with the soil pH.

Soil properties have been reported as a predominant factor responsible for the presence of fungal communities. For example, soil pH is the most critical factor in defining fungal community and activity through affecting pH homeostasis in the fungal cell or regulating the availability of soil nutrients [53]. A strong correlation has been demonstrated between available phosphorus and the abundance of the fungal phylum *Ascomycota* [54]. Another study reported that the population of *Ascomycota* in grassland soils increased with increasing available phosphorus [55]. Due to phosphorus being a limiting nutrient for plants, continued input of phosphorus fertilizer is required to increase and maintain plant production [56]. However, an inappropriate or high-rate use of fertilizer leads to serious problems associated with soil acidification [57]. The present study also found an increase in most of the HRM populations, and their heat resistance was likely due to an increase in available phosphorus of the soil and a decrease in soil pH. In agreement with this, Muneer et al. [58] reported that the relative abundance of *Ascomycota* was higher in soils with high NPK input compared to other nutrient management practices. Therefore, we suggested that soils with high acidity or a high phosphorus content, or both, had the potential for any HRM which has grown in that environment to produce highly thermal-resistant ascospores.

## 4. Conclusions

This study confirmed the presence of HRM ascospores in field soils where pineapples and sugarcane were cultivated. Almost all the HRM species exhibited high variability in thermal resistance across strains and some species did not produce heat-resistant ascospores. Again, in certain HRM species, the absence of ascospores on media does not mean that they do not produce heat-resistant ascospores in nature. The distribution of fungal genera and their ability to produce heat-resistant ascospores were affected by the physical and chemical properties of the soil. We observed that highly heat-resistant species were dominant in soil environments with a low pH or high phosphorus status, or both. Thus, soil management could be used to reduce the contamination of HRMs in raw fruit materials. To our knowledge, this is the first report on the relationship between the heat resistance of HRM ascospores and soil properties.

## Figures and Tables

**Table 1:** Physicochemical properties of soil used for isolation of HRMs.

Soil identification	Location	Province	Soil property				
			pH	CD	OM	AP	MC
KP1	Pineapple field	Kamphaeng Phet	4.80	61	0.2	12.4	11.65
KP2	Pineapple field	Kamphaeng Phet	5.70	100.7	0.28	2.8	7.39
KP3	Pineapple field	Kamphaeng Phet	4.69	189	0.58	8.8	10.05
KP4	Pineapple field	Kamphaeng Phet	6.67	73.3	0.84	3.6	8.24
CB1	Pineapple field	Chonburi	4.22	108	0.73	12.3	13.91
CB2	Pineapple field	Chonburi	4.67	50.4	0.39	134	11.9
CB3	Pineapple field	Chonburi	4.26	78.6	0.57	67.1	12.86
CB4	Pineapple field	Chonburi	5.00	108.3	0.6	15	8.95
CB5	Pineapple field	Chonburi	4.42	55.9	0.48	8.4	11.88
SK1	Sugarcane field	Sa Kaeo	5.73	28.35	0.23	2.4	14.5
PB1	Sugarcane field	Prachin Buri	6.20	135.2	0.68	38.3	15.19
PB2	Sugarcane field	Prachin Buri	5.49	61	0.27	4.0	12.86

KP: Kamphaeng Phet province, CB: Chonburi province, SK: Sa Kaeo province, PB: Prachin Buri province, CD: conductivity, OM: organic matter, AP: available phosphorus, and MC: moisture content.

## Data Availability

The data used in this study are available from the author on request.
